# Sustainable Effects of Human Dental Pulp Stem Cell Transplantation on Diabetic Polyneuropathy in Streptozotocine-Induced Type 1 Diabetes Model Mice

**DOI:** 10.3390/cells10092473

**Published:** 2021-09-18

**Authors:** Masaki Hata, Maiko Omi, Yasuko Kobayashi, Nobuhisa Nakamura, Megumi Miyabe, Mizuho Ito, Tasuku Ohno, Yuka Imanishi, Tatsuhito Himeno, Hideki Kamiya, Jiro Nakamura, Hitoshi Miyachi, Shogo Ozawa, Ken Miyazawa, Akio Mitani, Toru Nagao, Shigemi Goto, Jun Takebe, Tatsuaki Matsubara, Keiko Naruse

**Affiliations:** 1 Department of Removable Prosthodontics, School of Dentistry, Aichi Gakuin University, Nagoya 464-8651, Japan; hata@dpc.agu.ac.jp (M.H.); omim0611@gmail.com (M.O.); ag183d02@dpc.agu.ac.jp (Y.I.); ozawa@dpc.agu.ac.jp (S.O.); takebej@dpc.agu.ac.jp (J.T.); 2 Department of Internal Medicine, School of Dentistry, Aichi Gakuin University, Nagoya 464-8651, Japan; totoko721@hotmail.com (Y.K.); nnaka@dpc.agu.ac.jp (N.N.); mmiyabe@dpc.agu.ac.jp (M.M.); i-mizuho@dpc.agu.ac.jp (M.I.); matt@dpc.agu.ac.jp (T.M.); 3 Department of Periodontology, School of Dentistry, Aichi Gakuin University, Nagoya 464-8651, Japan; tasuku@dpc.agu.ac.jp (T.O.); minita@dpc.agu.ac.jp (A.M.); 4 Division of Diabetes, Department of Internal Medicine, Aichi Medical University School of Medicine, Nagakute 480-1195, Japan; himeno.tatsuhito.869@mail.aichi-med-u.ac.jp (T.H.); hkamiya@aichi-med-u.ac.jp (H.K.); nakamura.jirou.574@mail.aichi-med-u.ac.jp (J.N.); 5 Department of Maxillofacial Surgery, School of Dentistry, Aichi Gakuin University, Nagoya 464-8651, Japan; hitomiya@dpc.agu.ac.jp (H.M.); tnagao@dpc.agu.ac.jp (T.N.); 6 Department of Orthodontics, School of Dentistry, Aichi Gakuin University, Nagoya 464-8651, Japan; miyaken@dpc.agu.ac.jp (K.M.); shig@dpc.agu.ac.jp (S.G.)

**Keywords:** human dental pulp stem cells (hDPSCs), diabetic polyneuropathy, hDPSC-conditioned medium (hDPSC-CM), regenerative medicine

## Abstract

Dental pulp stem cells (DPSCs) are suitable for use in regenerative medicine. Cryopreserved human DPSCs (hDPSCs) ameliorate diabetic polyneuropathy, and the effects of hDPSC transplantation are related to VEGF and NGF secretion. This study evaluated the long-term effects of a single transplantation of hDPSCs on diabetic polyneuropathy. hDPSCs were obtained from human third molars extracted for orthodontic treatment, which were then transplanted into the unilateral hindlimb skeletal muscles 8 weeks after streptozotocin injection in nude mice. The effects of hDPSC transplantation were analyzed at 16 weeks post-transplantation. DPSC transplantation significantly improved delayed nerve conduction velocity, decreased blood flow, and increased sensory perception thresholds. Furthermore, the hDPSC-conditioned medium promoted the neurite outgrowth of dorsal root ganglion neurons. In conclusion, the therapeutic effects of hDPSC transplantation with a single injection last for prolonged periods and may be beneficial in treating long-term diabetic polyneuropathy.

## 1. Introduction

Cell therapy has the potential to treat injured or diseased tissues [1]. The homing and migration of transplanted stem cells are important for the ongoing replacement of mature cells and the regeneration of damaged tissues [2,3]. Multipotential differentiation of stem cells such as mesenchymal stem cells offer prospects for cell replacement where there is damaged tissue. However, previous studies revealed that many of the transplanted cells disappeared from the transplanted site after the studied period [4,5]. Furthermore, the resolution of adult tissue damage is a complex process unlikely to be solved by the transplanted stem cell itself [6]. Since transplanted cells produce abundant secretomes such as angiogenic factors, cytokines, and extracellular vesicles, these secretomes are considered to play a crucial role in tissue repair.

Dental pulp stem cells (DPSCs) in the dental pulp cavity are mesenchymal stem cells with a high proliferative ability and multilineage potential, which are retained after cryopreservation [7,8,9,10]. DPSCs also have immunomodulatory properties [11]. Since DPSCs can be easily obtained from teeth extracted during orthodontic therapy, they are considered appropriate for cell therapy, especially with regard to autologous transplantation [12,13]. The therapeutic effects of DPSC transplantation have been reported in several diseases such as Parkinson’s disease, spinal cord injury, myocardial infarction, liver fibrosis, and diabetic polyneuropathy (DPN) [9,14,15,16,17].

DPN is characterized by its early onset and the highest morbidity among the microvascular complications of diabetes and affects those with both type 1 and type 2 diabetes [18]. Symptoms of DPN vary such as spontaneous pain, hyperalgesia, and diminished sensation [19]. The pathogenesis of DPN includes degeneration of nerve fibers and reduced nerve blood flow. Tight glycemic control is important to slow the progress of DPN; however, this does not stop DPN progression [20]. Furthermore, multifactorial intervention that targets hyperglycemia, hypertension, and dyslipidemia failed to reduce the risk of DPN in patients with type 2 diabetes [21]. Therefore, novel therapeutic treatment is needed for DPN. Considering the pathophysiological factors of DPN, treatment with neuroprotective and angiogenic effects would be suitable.

Transplantation of human DPSCs (hDPSCs) ameliorated DPN in a type 1 diabetes mouse model, and the effects of transplantation were related to VEGF and NGF secretion [22]. Improvements in nerve conduction velocity, nerve blood flow, and sensory thresholds were assessed 4 weeks post-transplantation. However, it is not clear whether the effect of hDPSC transplantation can be maintained for a longer period. This paper evaluated the long-term effects of a single DPSC transplantation for DPN using a streptozotocin-induced type 1 diabetes mouse model.

## 2. Materials and Methods

### 2.1. Isolation of hDPSCs from Human Impacted Third Molars

**Figure 1:**
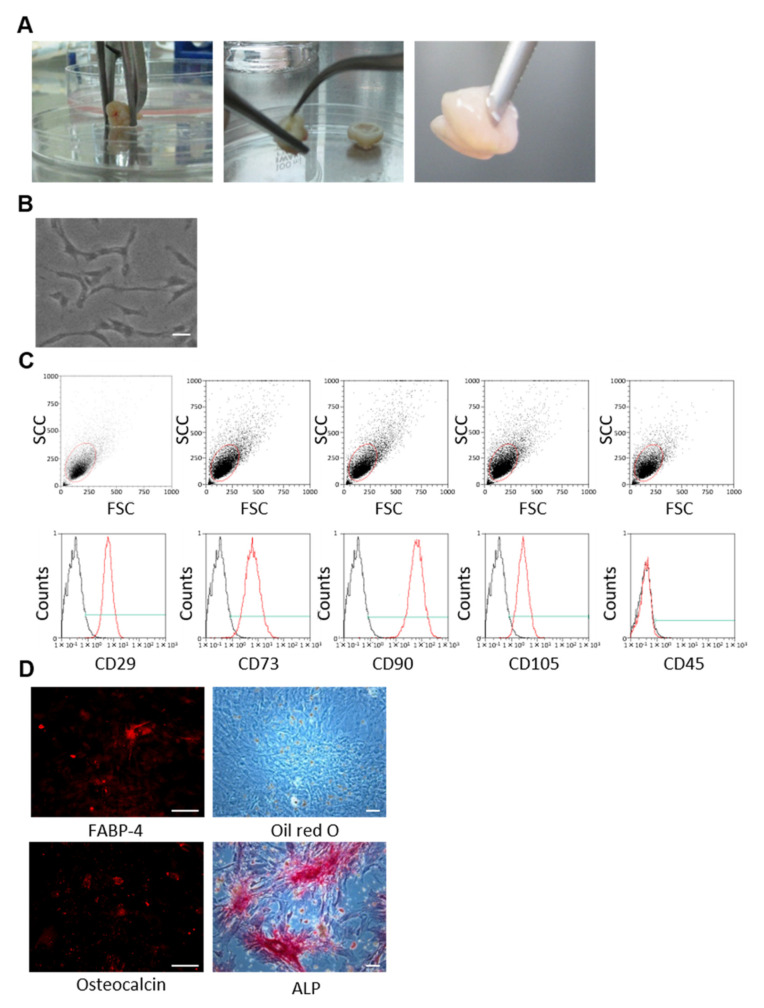
Identification of hDPSCs. (**A**) The dental pulp tissue was collected from third molars with forceps. (**B**) Observation of cultured hDPSCs with a phase-contrast microscope. Scale bar = 100 µm. (**C**) Surface marker expression, as analyzed by flow cytometry. Red circles: gated cells. Red lines: stained with specific antibodies. Black lines: isotype controls. Green lines: positive areas. Cell number: 1×10^5^ /mL. (**D**) Adipogenic differentiation detected by FABP-4 and Oil Red O staining. Osteogenic differentiation was detected by osteocalcin and ALP staining. Scale bar = 100 µm.

Human impacted third molars were obtained from 4 adults (13–23 years of age) by tooth extraction for orthodontics treatment at Aichi Gakuin University, with written informed consent obtained from each patient. Dental pulp tissue was extracted from the teeth (Figure 1A), and hDPSCs were collected and cultured, as previously described [23]. The cells were cryopreserved with CELL BANKER (Amsbio, Cambridge, MA, USA) at passage 3 and saved in a freezer. The cryopreserved hDPSCs were thawed and re-expanded for subsequent experiments. The study was conducted according to the guidelines of the Declaration of Helsinki, and approved by the Ethics Committee of the School of Dentistry, Aichi Gakuin University (AGU-142; 4 November 2008).

### 2.2. Flow Cytometrical Analyses of hDPSCs

Passage 3 hDPSCs were analyzed via fluorescence-activated cell sorting (MACS Qunat analyzer; Miltenyi Biotec, Bergisch Gladbach, Germany). Briefly, the cells were incubated with PE-conjugated mouse monoclonal antibodies against mouse CD29, CD73, CD90, and CD105 (Becton Dickinson, Franklin Lakes, NJ), and with FITC-conjugated mouse monoclonal antibodies against mouse CD45. Isotype-identical antibodies served as controls. The data were analyzed using MACS Quantify software Ver2.5 (Miltenyi Biotec) [22].

### 2.3. Adipogenic and Osteogenic Differentiation of hDPSCs

Adipogenic and osteogenic differentiation was performed according to the manufacturer’s instructions (R&D Systems, Minneapolis, MN, USA). To detect adipogenic differentiation, hDPSCs were stained with Oil Red O (Polysciences, Warrington, PA, USA) and fatty acid-binding protein-4 (FABP-4; R&D Systems). Osteogenic differentiation detection was performed by staining hDPSCs with alkaline phosphatase (ALP; Millipore, Darmstadt, Germany) and osteocalcin (R&D Systems).

### 2.4. Preparation of hDPSC-Conditioned Medium (hDPSC-CM)

When hDPSCs reached 70% confluence in 10 cm dishes, they were washed with PBS and maintained in Dulbecco’s modified Eagle’s medium (DMEM) containing penicillin and streptomycin. After 12 and 24 h, the culture media were collected and concentrated 10 times using 3 kDa centrifugal filters (Amicom Ultra-15, Nihon Millipore, Tokyo, Japan). The obtained hDPSC-CM was then frozen at −20 °C until use.

### 2.5. The Neurite Outgrowth of Primary Cultures of Mouse Dorsal Root Ganglion (DRG) Neurons

DRG neurons were obtained from 5-week-old male C57BL/6 mice (Chubu Kagakushizai, Nagoya, Japan), as previously described [24]. Briefly, DRG neurons were first separated using 0.125% collagenase (Wako Pure Chemical, Osaka, Japan) and trypsin (Sigma-Aldrich, St. Louis, MO, USA). After separation, they were diluted with a medium comprising F12 medium (Invitrogen, Carlsbad, CA), 10 nM glucose, and 30 nM selenium and seeded on glass coverslips coated with poly-L-lysine (Sigma-Aldrich). The neurons were then cultured for 24 h in serum-free F12 medium with or without hDPSC-CM. To evaluate neurite outgrowth, DRG neurons were immunostained with rabbit polyclonal anti-neurofilament heavy-chain antibody (1:5000, Nihon Millipore) and visualized with Alexa Fluor-594-coupled goat anti-rabbit antibody (Invitrogen). The immunoreaction was visualized using a fluorescence camera (DP70; Olympus Optical, Tokyo, Japan) under a fluorescence microscope (BX51; Olympus Optical). Neurite outgrowth was observed in 10–20 neurons per coverslip and evaluated using a computational image analysis system, as previously described (Angiogenesis Image Analyzer Ver 2; KURABO Industries, Osaka, Japan) [24].

### 2.6. Induction of Type 1 Diabetes

Six-week-old BALB/cAJcl-nu/nu male nude mice were obtained from Chubu Kagakushizai. Type 1 diabetes was induced by an intraperitoneal injection of streptozotocin (STZ; 150 mg/kg; Sigma Chemical, St. Louis, MO, USA) [25]. Mice with blood glucose levels of >14 mmol/L were identified as diabetic and were used in subsequent experiments. Age-matched male nude mice were used as control animals. The mice were housed in a room maintained at a controlled temperature (24 ± 1.0 °C) and with a 12 h light/dark cycle and were fed standard laboratory mouse chow along with water ad libitum. The experimental protocols were conducted according to the regulations for animal experiments at Aichi Gakuin University and were approved by the Institutional Animal Care and Use Committees of Aichi Gakuin University (AGU-59; 25 August 2009).

### 2.7. hDPSC Transplantation into Nude Mice

Eight weeks after the induction of diabetes in the mice, 1 × 10^5^ hDPSCs/limb in 0.2 mL of saline were injected at 10 separate sites in the unilateral hindlimb muscle. The vehicle (saline) was injected into the hindlimb muscle on the contralateral side as a control. Four and sixteen weeks post-transplantation, the following measurements were performed: sciatic nerve conduction velocity, blood flow of sciatic nerve, and current perception threshold.

#### 2.7.1. Sciatic Nerve Conduction Velocity

Mice were anesthetized with mixed anesthetic agents (medetomidine hydrochloride, midazolam, and butorphanol tartrate) and placed on a heated pad in a room maintained at 25 °C; the near-nerve temperature was maintained at 37 °C using a BAT-12 multipurpose thermometer (Bioresearch Co., Nagoya, Japan). The sciatic nerve motor nerve conduction velocity (MNCV) between the ankle and the sciatic notch was determined using Neuropak NEM-9400 (Nihon-Koden, Osaka, Japan). The sciatic nerve sensory nerve conduction velocity (SNCV) was measured between the knee and the ankle using retrograde stimulation.

#### 2.7.2. Blood Flow of the Sciatic Nerve

The blood flow of the sciatic nerve was measured by laser Doppler flowmetry (FLO-N1; Omegawave Inc, Tokyo, Japan). To measure the sciatic nerve blood flow (SNBF), the thigh skin of an anesthetized mouse was cut along the femur, and an incision was carefully performed through the fascia to expose the sciatic nerve. The blood flow was measured by a laser Doppler probe placed 1 mm above the nerve. During measurement, the muscle was placed on a heated pad in a room maintained at 25 °C to ensure a constant rectal temperature of 37 °C.

#### 2.7.3. Current Perception Threshold

To evaluate the nociceptive threshold, the current perception threshold (CPT) was measured 16 weeks post-transplantation using a CPT/LAB neurometer (Neurotron, Denver, CO, USA). The electrodes for stimulation were attached to plantar surfaces. Each mouse was kept in a Ballman cage (Natsume Seisakusho, Tokyo, Japan) suitable for light restraint in the awake state. Transcutaneous nerve stimuli consisting of three sine wave pulses (at 5, 250, and 2000 Hz) were applied to the plantar surfaces. The intensity of each stimulation was gradually increased automatically. The minimum intensity at which each mouse withdrew its paw was defined as the CPT, which was reported as the mean of the values obtained from the fourth and fifth measurements.

#### 2.7.4. Location of Transplanted hDPSCs in the Hindlimb Skeletal Muscles

Sixteen weeks after the transplantation, the mice were euthanized via CO_2_ inhalation. The gastrocnemius skeletal muscles were collected and fixed with 4% paraformaldehyde. The muscles were embedded in a specific immersing solution (SCMM) in liquid nitrogen and cooled isopentane. Afterwards, the frozen samples were embedded in a mounting medium and cut into 5 µm sections using adhesive film. Then, the sections were incubated with primary antibodies (anti-human nuclei antibody; Millipore). Human nuclei were confirmed using the Zenon Alexa Fluor 488 Mouse IgG1 Labeling Kit (Thermo Fisher Scientific, Waltham, MA). The sections were observed using fluorescence microscopy (Leica AF6000LX, Leica Microsystems, Wetzlar, Germany).

#### 2.7.5. Human Gene Expression in the Hindlimb Skeletal Muscles

Total RNA was taken from frozen samples of gastrocnemius skeletal muscles using TRIzol Reagent (Invitrogen) according to the manufacturer’s instructions. ReverTra Ace (Toyobo, Osaka, Japan) synthesized cDNA from 1 µg of the RNA. Primers for human and mouse β-actin were purchased from Taqman Gene Express ion Assays (Applied Biosystems, Foster City, CA, USA). Polymerase chain reaction (PCR) products were visualized by agarose gel (Wako, Osaka, Japan) electrophoresis with ethidium bromide staining.

### 2.8. Statistical Analysis

Data are expressed as the mean ± standard error of the mean (SEM). Statistical analysis was performed using Student’s t-test for two groups and one-way ANOVA with Bonferroni correction for multiple comparisons. Differences were considered significant at *p* < 0.05.

## 3. Results

### 3.1. Identification of hDPSCs

hDPSCs cultured on a plastic dish exhibited a typical spindle-shaped morphology (Figure 1B). Flow cytometry revealed that hDPSCs expressed CD29, CD73, CD90, and CD105 on their surface but not CD45 (Figure 1C). To confirm their differentiation capability, hDPSCs were cultured in adipogenic and osteogenic induction media. The hDPSCs differentiated into adipocytes, as detected by FABP-4 and Oil Red O staining, and into osteoblasts, as detected by ALP and osteocalcin staining, 3 weeks after induction (Figure 1D).

### 3.2. hDPSC-CM Promoted Neurite Outgrowth of DRG Neurons

**Figure 2:**
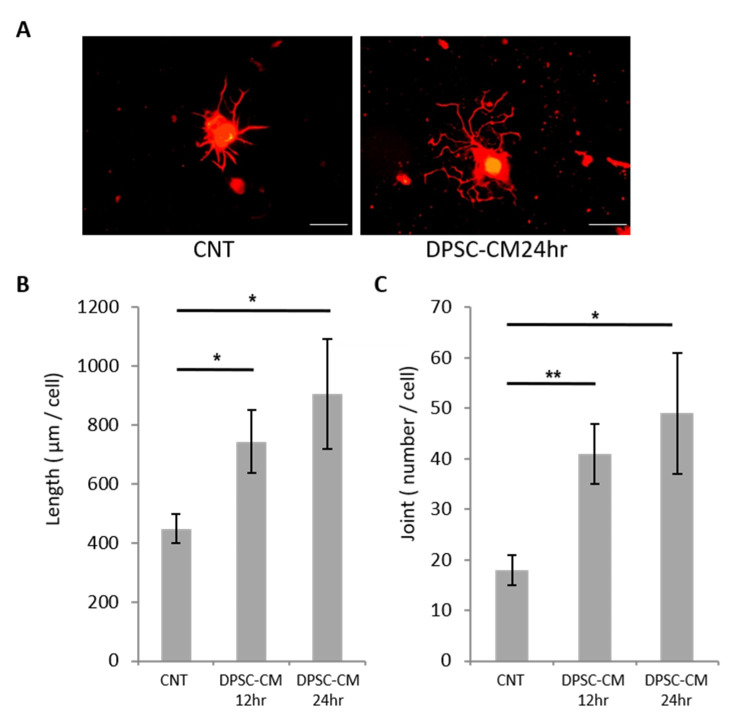
Neurite outgrowth of DRG neurons with hDPSC-CM. (**A**) Representative fluorescence micrograph of DRG neurons stained for neurofilaments in culture with hDPSC-CM after 24 h. Scale bar = 50 µm. Measurements of total neurite length (**B**) and joint number of neurites (**C**). The results are expressed as the mean ± SEM (*n* = 11). * *p* < 0.05, ** *p* < 0.01. CNT: control, DPSC-CM: DPSC-conditioned medium.

To investigate whether hDPSCs can affect DRG neurite outgrowth, we measured the total length (TL) and joint number (JN) of neurites among DRG neurons cultured with or without hDPSC-CM. The neurite outgrowth of DRG neurons increased in the presence of hDPSC-CM (Figure 2A). Quantitative analyses revealed that hDPSC-CM significantly increased the TL and JN at 12 and 24 h (Figure 2B,C).

### 3.3. Body Weight and Blood Glucose Level

**Figure 3:**
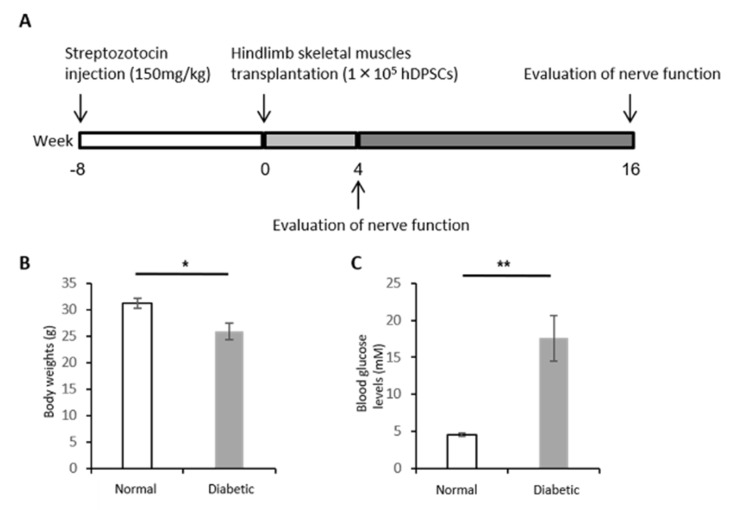
Experimental animal protocol. (**A**) hDPSCs were transplanted into unilateral hindlimb skeletal muscles 8 weeks after streptozotocin injection, and physiological assessments were performed 4 and 16 weeks after hDPSC transplantation. (**B**) Body weights. (**C**) Blood glucose concentrations. The results are expressed as the mean ± SEM (*n* = 6). * *p* < 0.05, ** *p* < 0.01.

At the end of the experiments, 24 weeks after STZ injection and 16 weeks after hDPSC transplantation, diabetic mice showed a significant decrease in body weight (normal mice: 31.3 ± 1.0 g; diabetic mice: 25.9 ± 1.6 g; *p* < 0.05) and a significant increase in blood glucose levels (normal mice: 4.6 ± 0.2 mM; diabetic mice: 17.6 ± 3.1 mg/dL; *p* < 0.01) (Figure 3A–C).

### 3.4. hDPSC Transplantation Improved the MNCV and SNCV in Diabetic Mice

**Figure 4:**
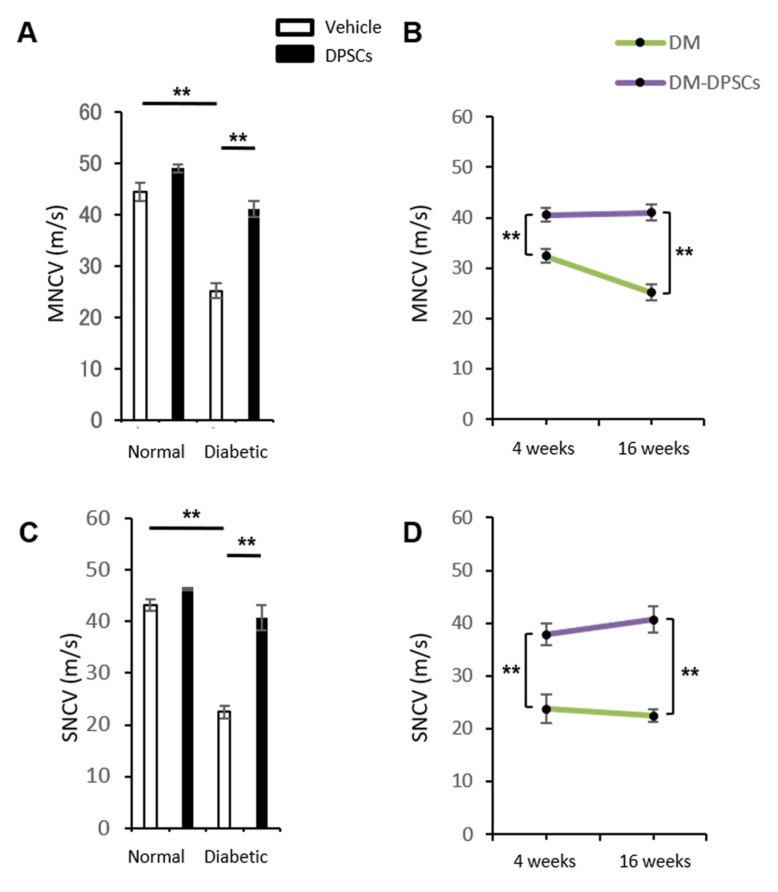
Nerve conduction velocity. (**A**) Sciatic motor nerve conduction velocity at 16 weeks after hDPSC transplantation. (**B**) Sciatic motor nerve conduction velocity at 4 and 16 weeks after hDPSC transplantation in diabetic mice. (**C**) Sciatic sensory nerve conduction velocity at 16 weeks after hDPSC transplantation. (**D**) Sciatic sensory nerve conduction velocity at 4 and 16 weeks after hDPSC transplantation in diabetic mice. The results are expressed as the mean ± SEM (*n* = 4). ** *p* < 0.01. DM: diabetes mellitus.

The MNCV and SNCV in the vehicle-injected side of diabetic mice significantly decreased compared to normal mice at 24 weeks after STZ injection (*p* < 0.01). The MNCV and SNCV of diabetic mice were significantly restored in the hDPSC-injected side at 16 weeks post-transplantation (*p* < 0.01) (Figure 4A,C). The therapeutic effects of hDPSC transplantation on the MNCV and SNCV were observed at 4 weeks post-transplantation, and these effects were maintained until 16 weeks post-transplantation (Figure 4B,D). However, hDPSC transplantation did not affect the MNCV or SNCV in normal mice.

### 3.5. hDPSC Transplantation Increased the Sciatic Nerve Blood Flow in Diabetic Mice

**Figure 5:**
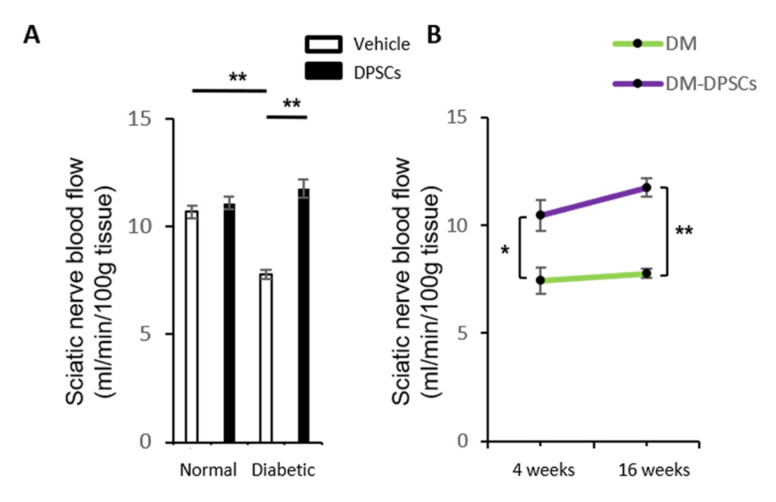
Sciatic nerve blood flow. (**A**) Sciatic nerve blood flow at 16 weeks after hDPSC transplantation. (**B**) Sciatic nerve blood flow at 4 and 16 weeks after hDPSC transplantation in diabetic mice. The results are expressed as the mean ± SEM (*n* = 4). * *p* < 0.05, ** *p* < 0.01. DM: diabetes mellitus.

The sciatic nerve blood flow in the vehicle-injected side of diabetic mice significantly decreased compared to normal mice (*p* < 0.01). hDPSC transplantation significantly augmented the sciatic nerve blood flow in the hDPSC-injected side of diabetic mice at 16 weeks post-transplantation (*p* < 0.01) (Figure 5A). hDPSC transplantation also maintained the sciatic nerve blood flow equivalent to normal mice in the hDPSC-injected side of diabetic mice. These effects were confirmed from 4 to 16 weeks after hDPSC transplantation (Figure 5B). Again, hDPSC transplantation caused no significant changes in the SNBF of normal mice.

### 3.6. hDPSC Transplantation Improved the Current Perception Thresholds in Diabetic Mice

**Figure 6:**
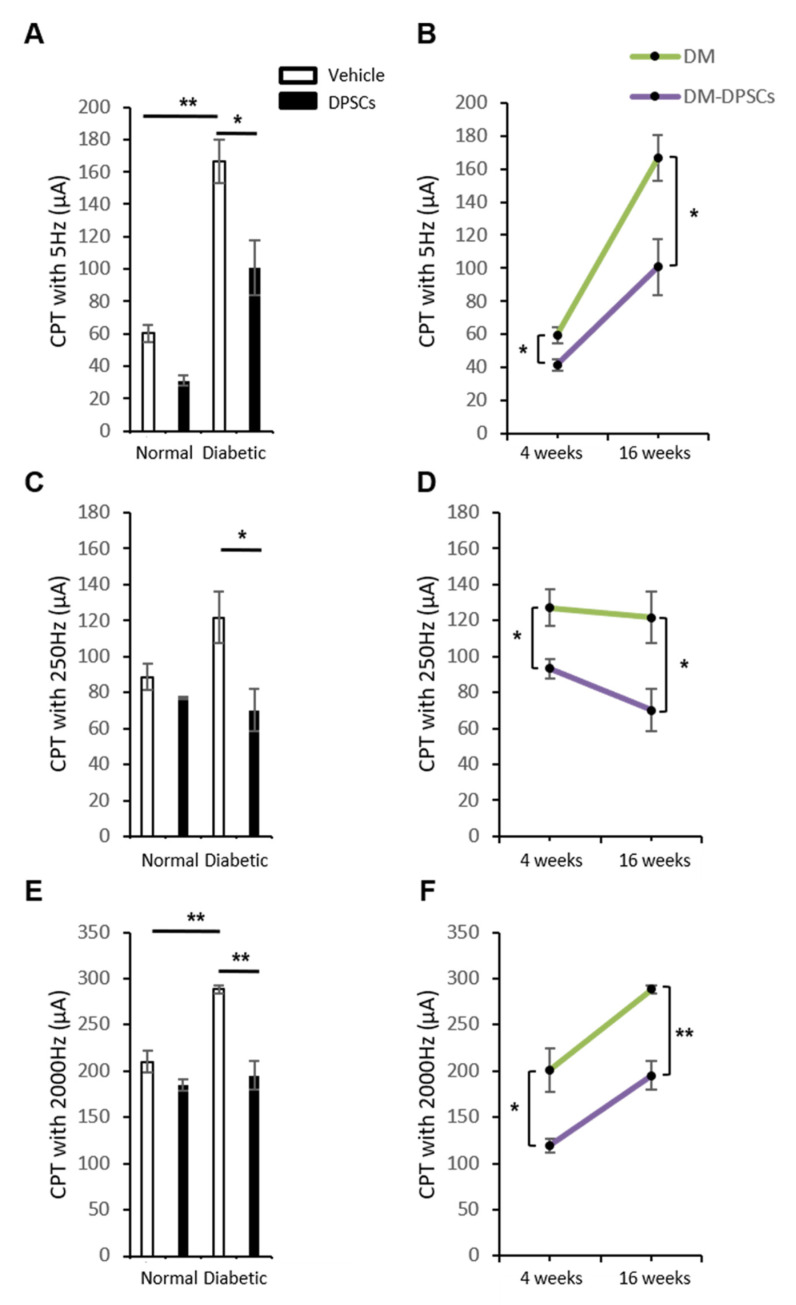
Sensory nerve functions. Measurements of current perception thresholds (CPTs) at 5, 250, and 2000 Hz, which demonstrated the thresholds of the C fiber, Aδ fiber, and Aβ fiber of peripheral nerves. (**A**) CPTs at 5 Hz at 16 weeks after hDPSC transplantation. (**B**) CPTs at 5 Hz at 4 and 16 weeks after hDPSC transplantation in diabetic mice. (**C**) CPTs at 250 Hz at 16 weeks after hDPSC transplantation. (**D**) CPTs at 250 Hz at 4 and 16 weeks after hDPSC transplantation in diabetic mice. (**E**) CPTs at 2000 Hz at 16 weeks after hDPSC transplantation. (**F**) CPTs at 2000 Hz at 4 and 16 weeks after hDPSC transplantation in diabetic mice. The results are expressed as the mean ± SEM (*n* = 4). * *p* < 0.05, ** *p* < 0.01. DM: diabetes mellitus.

We assessed sensory functions with the current perception threshold (CPT). CPTs at 5, 250, and 2000 Hz, which demonstrated the thresholds of the C fiber, Aδ fiber, and Aβ fiber, respectively, of peripheral nerves significantly increased compared to normal mice, representing hypoalgesia in diabetic mice. Sixteen weeks after hDPSC transplantation, these deficits in sensation significantly improved in diabetic mice compared to the vehicle-injected side of diabetic mice (5 and 250 Hz: *p* < 0.05; 2000 Hz: *p* < 0.01) (Figure 6A,C,E). The therapeutic effects of hDPSC transplantation in diabetic mice could be observed at 4 and 16 weeks post-transplantation (Figure 6B,D,F).

### 3.7. Location of the hDPSCs 16 Weeks after Transplantation

**Figure 7:**
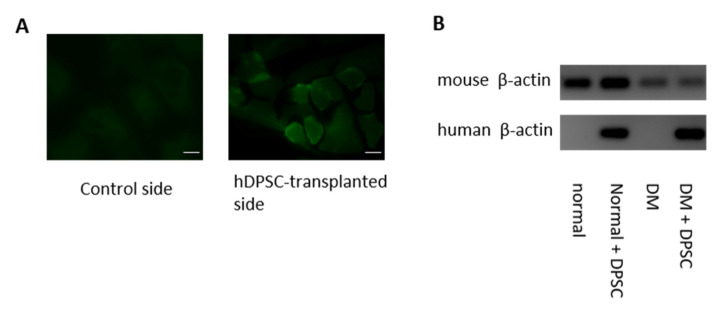
Location of transplanted hDPSCs in the gastrocnemius muscles. (**A**) Sixteen weeks after hDPSC transplantation in the hindlimb skeletal muscles, the transplanted cells were stained with anti-human nuclei antibody. Bar = 25 µm. (**B**) The human β-actin mRNA expression was evaluated by real-time quantitative polymerase chain reaction. The products were visualized by agarose gel electrophoresis with ethidium bromide staining. DM: diabetes mellitus.

The locations of the transplanted hDPSCs were assessed by immunohistological staining with anti-human nuclei antibody. Some of the hDPSCs transplanted into the skeletal muscles were present around the muscle bundles at 16 weeks after transplantation. Human nuclei on the control side were not detected in either normal or diabetic mice (Figure 7A). As shown in Figure 7B, the hDPSC-transplanted side of the hindlimb skeletal muscles expressed human β-actin mRNA in both normal and diabetic mice. No human genes were expressed on the control side.

## 4. Discussion

The therapeutic efficacy of hDPSC transplantation by a single injection was maintained for 16 weeks post-transplantation with DPN treatment in diabetic nude mice. The decreased MNCV, SNCV, and SNBF in diabetic mice were ameliorated by hDPSC transplantation at 16 weeks post-transplantation. Hypoalgesia at the Aβ, Aδ, and C fibers also recovered post-transplantation.

DPSCs are easy to collect from dental pulp tissue [26]. They maintain their proliferative and differential abilities after cryopreservation, and thus they are considered suitable for cell therapy [27]. Studies have reported the clinical application of hDPSCs in Alzheimer’s disease, corneal reconstruction, and cerebral ischemia [28,29,30]. hDPSC transplantation into skeletal muscles ameliorates diabetic polyneuropathy at 4 weeks post-transplantation [22]. In this study, a single transplantation of hDPSCs into diabetic mice ameliorated the sciatic nerve motor nerve, sensory nerve, sciatic nerve blood flow, and CPTs, even after 16 weeks of transplantation.

One of the therapeutic mechanisms of hDPSC transplantation for DPN may be the continuous secretion of multipotent factors, including angiogenic and neurotrophic factors, by transplanted cells. hDPSC-CM promotes the neurite outgrowth of DRG neurons. The administration of VEGF- and NGF-neutralizing antibodies inhibits the effects of hDPSC transplantation on the MNCV and SNCV [22]. Our previous study revealed the critical role of the secreted factors from DPSCs [31]. We performed a direct comparison between DPSC transplantation and the administration of DPSC-secreted factors (DPSC-SFs) on diabetic polyneuropathy and demonstrated that both DPSC transplantation and DPSC-SF administration significantly ameliorated diabetic polyneuropathy, while there was no difference between the results for DPSCs and DPSC-SFs.

On the other hand, some of the transplanted hDPSCs are located around muscle bundles and express human VEGF and NGF mRNA in transplanted skeletal muscles at 4 weeks post-transplantation [22]. Retaining transplanted cells at injected sites may have a positive effect on the cells themselves and surrounding cells, which may prolong the therapeutic effects. We confirmed that some of the transplanted hDPSCs were still localized around muscle bundles, and the expression of human β-actin mRNA was still found at 16 weeks post-transplantation. Further studies are required to elucidate further information on this issue.

The effects of hDPSC transplantation on DPN are maintained until 4 months post-transplantation in type 1 diabetes. To the best of our knowledge, this is the longest study to investigate the sustainability of the effects of stem cell transplantation on diabetic polyneuropathy in type 1 diabetes. Mao et al. demonstrated that single autologous bone marrow-derived mononuclear cell transplantation ameliorated refractory DPN for 36 months in type 2 diabetes patients [32]. Therefore, a single intramuscular injection of stem cells ameliorates DPN in both type 1 and type 2 diabetes for prolonged periods.

The advantages of cell therapy for DPN using hDPSCs are as follows. First, young hDPSCs can be easily obtained without further invasion from extracted teeth at an early age, which makes it possible to resolve stem cell dysfunction by aging and diabetes. Second, hDPSCs can be easily expanded and retain their proliferative ability and multilineage potential after cryopreservation. An optimal dose and time point for DPSC transplantation needs to be investigated because therapeutic effects interact with the local microenvironment under inflammatory conditions and tissue damage [33].

In conclusion, hDPSC transplantation revealed that the recovery of nerve conduction velocity, blood flow, and sensory nerve fibers lasts until 16 weeks post-transplantation. Secreted factors from hDPSCs also have neurotropic action in DRG neurons. Therefore, hDPSCs may have an additional role to play in treating DPN in the future.
